# Peripheral Serotonin Regulates Maternal Calcium Trafficking in Mammary Epithelial Cells during Lactation in Mice

**DOI:** 10.1371/journal.pone.0110190

**Published:** 2014-10-09

**Authors:** Jimena Laporta, Kimberly P. Keil, Chad M. Vezina, Laura L. Hernandez

**Affiliations:** 1 Department of Dairy Science, University of Wisconsin-Madison, Madison, Wisconsin, United States of America; 2 Department of Comparative Biosciences, School of Veterinary Medicine, University of Wisconsin-Madison, Madison, Wisconsin, United States of America; University of Tennessee, United States of America

## Abstract

Lactation is characterized by massive transcellular flux of calcium, from the basolateral side of the mammary alveolar epithelium (blood) into the ductal lumen (milk). Regulation of calcium transport during lactation is critical for maternal and neonatal health. The monoamine serotonin (5-HT) is synthesized by the mammary gland and functions as a homeostatic regulation of lactation. Genetic ablation of tryptophan hydroxylase 1 (*Tph1*), which encodes the rate-limiting enzyme in non-neuronal serotonin synthesis, causes a deficiency in circulating serotonin. As a consequence maternal calcium concentrations decrease, mammary epithelial cell morphology is altered, and cell proliferation is decreased during lactation. Here we demonstrate that serotonin deficiency decreases the expression and disrupts the normal localization of calcium transporters located in the apical (PMCA2) and basolateral (CaSR, ORAI-1) membranes of the lactating mammary gland. In addition, serotonin deficiency decreases the mRNA expression of calcium transporters located in intracellular compartments (SERCA2, SPCA1 and 2). Mammary expression of serotonin receptor isoform 2b and its downstream pathways (PLCβ3, PKC and MAP-ERK_1/2_) are also decreased by serotonin deficiency, which might explain the numerous phenotypic alterations described above. In most cases, addition of exogenous 5-hydroxy-L-tryptophan to the *Tph1* deficient mice rescued the phenotype. Our data supports the hypothesis that serotonin is necessary for proper mammary gland structure and function, to regulate blood and mammary epithelial cell transport of calcium during lactation. These findings can be applicable to the treatment of lactation-induced hypocalcemia in dairy cows and can have profound implications in humans, given the wide-spread use of selective serotonin reuptake inhibitors as antidepressants during pregnancy and lactation.

## Introduction

During lactation, calcium secretion into milk by the mammary epithelial cells (MEC) increases dramatically. Regulation of maternal calcium levels during lactation, achieved through molecular and physiological adjustments in calcium homeostasis, is critical to sustain milk synthesis and to satisfy maternal calcium needs [1]. Impaired calcium homeostasis during the early periparturient period causes hypocalcemia in bovine and canine species [2]–[4]. In particular, hypocalcemia is one of the most common metabolic diseases of dairy cattle [5] with profound negative economic and welfare implications to the dairy industry [2], [3], [6].

The mammary gland is a highly adapted organ that consists of a complex network of cell types that can respond to different molecular and endocrine signals. Particularly during lactation, the mammary gland drives calcium homeostasis. MECs have developed a network of transporters and pumps that enables the transport of calcium from the blood into the milk [7]. The *calcium sensing receptor* (CaSR) and *calcium release-activated calcium channel protein 1* (ORAI-1) are responsible for moving calcium from the circulation into the MEC, and the *plasma membrane Ca2+ ATPase 1 and 2* (PMCA1, 2) are involved in regulation of calcium fluxes in MEC and the pumping of calcium into the milk, respectively. In intracellular compartments, the *sarcoplasmic endoplasmic Ca^2+^ ATPase 2* (SERCA2) stores Ca within the rough endoplasmic reticulum, and the *secretory pathway Ca^2+^ ATPase 1 and 2* (SPCA1 and 2) are involved in pumping calcium in and out of the Golgi apparatus. The *sodium/calcium exchanger 1* (NCX1) participates in MECs trans-epithelial calcium transport, however its exact localization in the MEC is not clear [7]–[12].

Lactation induces the expression on ‘non-classical’ hormones and factors produced locally by the MECs. The monoamine serotonin (5-HT) impact milk protein gene expression, tight junction permeability, calcium and glucose homeostasis during lactation [13]–[19]. Tryptophan hydroxylase 1 (TPH1) is the rate-limiting enzyme in 5-HT synthesis and converts L-tryptophan into 5-hydroxy-L-tryptophan (5-HTP) [13], which is then converted to serotonin, by aromatic l-amino acid decarboxylase. serotonin exerts its actions by signaling through more than 15 receptor subtypes found on various tissues [20]. In lactating rat and mouse dams, serotonin induces mammary gland synthesis and secretion of parathyroid hormone related protein (PTHrP), which activates bone osteoclasts and mobilizes calcium reserved into the circulation of the dam [19], [21], [22]. In addition, circulating serotonin concentrations in dairy cattle on d 1 of lactation is positively correlated with circulating calcium and PTHrP concentrations, and negatively correlated with the incidence of hypocalcemia, therefore supporting serotonin involvement in calcium homeostasis [23].

Here, we tested the hypothesis that serotonin is required for the appropriate expression and localization of calcium transporters in the lactating mammary gland. We used *Tph1* deficient mice to reduce peripheral 5HT synthesis. We also explore plausible downstream pathways that might be involved in the mechanism(s) by which serotonin regulates mammary gland function during lactation. Understanding how serotonin affects calcium transport within the MECs can have therapeutic implications for treatment of lactation-induced hypocalcemia in dairy cattle, and could also have implications for the treatment of depression in humans during lactation.

## Materials and Methods

### Ethic Statement

All experiments were performed under protocols approved by the Research Animal Care and Use Committee at the University of Wisconsin-Madison. The protocol number assigned to Dr. Laura L. Hernandez for these experiments was A1473.

### Animal Handling and Experimental Design

Twenty-one pregnant female C57B6/J mice were used and maintained in a controlled environmental facility for biological research at the Animal Science Department, University of Wisconsin-Madison. Mice were maintained at a temperature of 25°C and humidity of 50%–60% controlled environment on a 12-h light/dark cycle with free access to food and water. Pregnant dams were randomly assigned to individual cages from day 15 of gestation until day 10 of lactation. Mice were assigned to 3 groups: group 1 consisted of *Tph1* deficient dams (*Tph1* ^−/−^, n = 7), group 2 consisted of *Tph1* ^−/−^ dams injected i.p. with 100 mg/kg of 5-HTP (*Tph1* ^−/−^ (5-HTP), n = 7) to rescue peripheral serotonin function, and group 3 consisted of wild-type dams (WT, n = 7). WT and *Tph1* ^−/−^ dams were injected daily with saline to control for the variable of stress due to injection. Injections began on d 15 of gestation and ended on d 10 of lactation when all mice and pups were euthanized. Litter size was standardized to 6 pups per dam, regardless of their sex, to control for prolactin surge due to number of pups nursing.

### Data and Sample Collection

Litter weights per dam and milk yield were recorded daily, with milk yield being estimated by the weigh-suckle-weigh method [24]. Dam weights were recorded at the beginning and end of the experiment. Serum samples were harvested from blood collected from the maxillary vein on d 1, d 5 and d 10 of lactation. On d 10 of lactation, which is the peak of lactation in female mice, all dams were euthanized and mammary gland number 4 was collected for RNA and total protein isolation. Tissue was stored at −80°C until used. The second mammary gland number 4 was fixed in 4% paraformaldehyde overnight at 4°C and then embedded in paraffin and sectioned (5 µm) for histological evaluation.

### Blood serotonin and Calcium Assays

Serum serotonin concentrations were determined by ELISA (Enzo Life Sciences, #ADI-900-175), according to the manufacturer’s instructions. Serum was diluted 1∶50 to detect serotonin concentrations within the parameters of the assay. The intra-assay CV was 2.6%. Total serum calcium concentrations were measured with a calcium assay kit (Cayman Chemical Company, #700550) according to the manufacturer’s instructions. The intra-assay CV was 3.4%.

### Mammary gland RNA extraction and Quantitative Real-time RT-PCR (qPCR)

Total RNA was isolated from bone and mammary gland tissue with the use of TRI-Reagent (Molecular Research) and was reverse transcribed (1 µg) to cDNA (Bio-Rad, #1708841). Quantitative RT-PCR (qPCR) was conducted with the CFX96 Touch Real-Time PCR Detection System (Bio-Rad). Reaction mixtures, cycling conditions were performed as previously described [19]. Amplification efficiencies of primers were evaluated and were within a range of 95% and 105% efficiency, and primer specificity was assessed by the presence of a single temperature dissociation peak. Primer sequences used are listed Table 1. Ribosomal protein S15 (RPS15) was used as the housekeeping gene, and analysis was conducted with the 2^−ΔΔCt^ method [25].

**Table 1:** Primers sequences for the studied genes quantified by real-time PCR.

Genes	Forward primer 5′→3′	Reverse primer 5′→3′
CaSR	TCTTGTGGAGTGGGTTCTCC	AAAACAGCAGGTGGGCTCT
NCX1	CCCAGGACCAGTATGCAGAT	CATGGTAGATGGCAGCAATG
Orai1	ACCCCACGAGCGCATGCATC	GCTTGGTGGGGCTTGGCTGT
PMCA1	AACGACTGGAGCAAGGAGAA	CCGTACTTCACTTGGGCAAT
PMCA2	ACGTATGGGGACACTGAAGC	TTGCCCAAAAATCTGTTTCC
RPS15	GTTGAAGGTCTTGCCGTTGT	TTGAGAAAGGCCAAAAAGGA
SERCA2	TACTGACCCTGTCCCTGACC	CACCACCACTCCCATAGCTT
SPCA1	AGGCAGAAGAAGCACCAAAA	TAACCAGCCAACCAACATGA
SPCA2	CCTGTGCAACGAGAAACTGA	CCTGCAACGCCTTTATGATT
Htr2b	CAATCATCCTCCTCGATACCC	GAAGCCATCAGATCTACTTTAGCC

All primers were designed using Primer 3 [43] except for Orai1 that was obtained from Cross et al. [8]. All primers were run at an annealing temperature of 60°C and ribosomal protein 15 (RPS15) was used as the housekeeping gene.

### Mammary Gland Protein Isolation and Protein Assays

Protein was isolated from the mammary gland tissue using radioimmunoprecipitation assay buffer (RIPA) plus 10 µL/mL of Halt Protease and Phosphatase Inhibitors Cocktail (Thermo scientific #78441). Protein concentrations were determined with the bicinchoninic acid assay (Pierce Chemicals #23227).

Mammary gland concentration of serotonin was determined by ELISA (EIA kit; Enzo life Sciences, #ADI-900-175), using 50 µg of protein per sample analyzed. The intra-assay CV was 6.6%. Imunoblotting was performed and analyzed as described previously [18]. Briefly, mammary gland total protein extracts (25 µg) were separated by electrophoresis on a 10% SDS-polyacrylamide gel and transferred for 1.5 h at 50 V and 4°C onto polyvinylidene difluoride membrane (Millipore, #IPVH00010). Membranes were blocked with 5% BSA for 1.5 h and probed overnight at 4°C with 1∶5,000 β-actin (Sigma-Aldrich, #A5441), 1∶500 CaSR (Thermo Scientific, #MA1-934), 1∶1,500 ORAI-1 (Millipore, #AB9868), 1∶1,000 PMCA2 (Abcam, #ab3529), 1∶1,000 PKC (Anti PKC-PAN, Sigma-Aldrich, #SAB4502356), 1∶1,250 PLCβ3 (Thermo Scientific, #PA5-17772), 1∶1,500 ERK_1/2_ and 1∶5,000 pERK_1/2_ (Cell signaling # 9102 and 9101, respectively). Membranes were incubated for 1 h at room temperature with horseradish peroxidase-conjugated secondary antibodies (1∶5,000 donkey anti-rabbit IgG Santa Cruz #sc-2305 for Orai1, PMCA2, PKC and PLCβ3; 1∶10,000 and 1∶500 donkey anti-moue Santa Cruz #sc-2314 for β-actin and CaSR, respectively; and 1∶200 anti-rabbit Cell signaling #7074) and StrepTactin-HRP conjugate (Bio-Rad, #161-0381) for chemiluminescent detection. Protein bands were detected at multiple exposure times using Immun-StarTM WesternCTM Kit (BioRad, #170-5070) and visualized using the Chemidoc XRS system (BioRad, #1708070). Image processing and protein band quantification was done using QuantityOne (v.4.6.9) Analysis Software (BioRad).

### Mammary Gland Histology and Immunofluorescence

Sectioned mammary glands were stained with hematoxylin and eosin (H&E). Images were used to quantify alveoli number and diameter using ImageJ software (NIH Version 1.48a). Mammary gland slides were deparaffinized and processed for immunofluorescence. Cell proliferation was determined by immunolabeling with Ki67 antibody (Abcam, #5580, 1∶200) in combination with an epithelial cell marker (E-Cadherin, BD Biosciences, #610181, 1∶250). Secondary antibodies were incubated for 1 h at room temperature and diluted as follows: 1∶250, Alexa Fluor 594 Goat Anti-Rabbit IgG (Life Technologies, #A11012) and 1∶250, DyLight 488-conjugated Goat Anti-Mouse IgG (Jackson Immuno Research #115-487-003). Nuclei were visualized with 49, 6-diamidino-2-phenylindole (DAPI, 1∶1,000 in blue). Proliferating cell numbers were determined using ImageJ software.

Mammary sections were incubated overnight at 4°C with CaSR primary antibody (Thermo Scientific #MA1-16940, 1∶100) in combination with an apical membrane marker Mucin-1 (MUC1; Abcam #ab15481, 1∶50). Secondary antibodies against CaSR and MUC1 were incubated for 1 h at room temperature and diluted 1∶250 DyLight 549-conjugated Goat Anti-Mouse IgG (Jackson Immuno Research #115-507-1003), 1∶250 and 1∶500 DyLight 488-conjugated Anti-Rabbit IgG (Jackson Immuno Research #111-487-003), respectively. Mammary sections were incubated overnight at 4°C with PMCA2 primary antibody (Abcam, #ab3529, 1∶50) in combination with β-Cat to mark the basolateral membrane. Secondary antibodies were incubated for 1 h at room temperature, Alexa Fluor 594 Goat Anti-Rabbit IgG (Life Technologies, #A11012) was used against PMCA2 and diluted at 1∶250. Some mammary sections were incubated with ORAI-1 primary antibody (Sigma #4200273, 1∶100) and co-stained with MUC1 to mark the apical membrane. Secondary antibodies were incubated for 1 h at room temperature, DyLight 549-conjugated Goat Anti-Mouse IgG (Jackson Immuno Research #115-507-1003,) was used against ORAI-1 and diluted at 1∶250. Nuclei were visualized with 49, 6-diamidino-2-phenylindole (DAPI, 1∶1,000 in blue). All images were captured (40 X magnification) using a Zeiss Axio Vert. A1 fluorescent microscope and processed and merged using Q-capture pro 7 software.

### Statistical Analyses

Statistical analysis was conducted with Prism version 6.0 b (GraphPad Software). Gene and protein expression, alveoli size and number, dam body weight, blood serotonin and calcium, were analyzed using one-way ANOVA followed by Tukey’s post hoc test to test differences between the groups. If sample populations did not have equal variances, Welch’s correction was applied. Normality and outlier tests were performed using Shapiro-Wilk test and Grubbs test (ESD method), respectively. Litter weights and milk yield data were analyzed by two-way ANOVA with group, time, and the interaction between group and time serving as main effects. Multiple comparisons were tested with the Holm-Sidak method to detect differences between treatment groups. Differences between means were considered significant at *P*<0.05. All values are reported as means ± SEM.

## Results

### *Tph1* gene ablation does not affect dam and litter growth or dam milk yield

serotonin at high concentrations can cause mammary gland involution [26] potentially affecting milk yield and pup growth. Therefore, we first evaluated if *Tph1* gene ablation affected dam and litter weights, and milk yield. Dam body weight was similar between all group comparisons, both at the beginning of the experiment and on d10 of lactation (31.2±3.5 and 26.4±1.5 g average of all groups, respectively; *P*>0.24). Pup growth and dam milk yield/bout of nursing were affected by time (days of lactation, *P*<0.001), reflecting offspring’s normal growth pattern (7.7±0.24, 10.5±0.7, 16±1.5 g, mean ± SEM of three groups for d1, 5, and 10, respectively) and dam milk production (0.035±0.01, 0.25±0.03 and 0.42±0.9, mean ± SEM of milk yield/bout of nursing for all three groups) but there were no significant differences between groups (*P*>0.05) at any time point comparison.

### *Tph1* gene Ablation alters Mammary Epithelial Cell Morphology and Proliferation during Lactation

**Figure 1:**
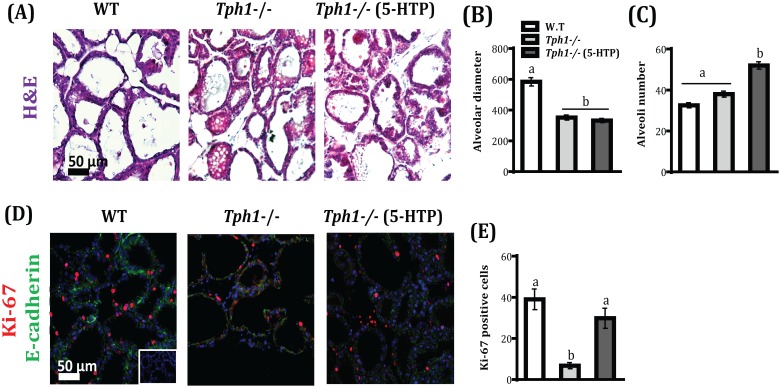
Tph1 gene ablation alter mammary epithelial cell morphology and cell proliferation on d 10 of lactation. Wild-type (WT) mouse dams were given saline and Tryptophan hydroxylase knock-out dams given either saline (*Tph1^−/−^*) or daily injections of exogenous 5-HTP (100 mg/kg; *Tph1^−/−^* (5-HTP)), from pregnancy d20 to lactation d10. (A) Mammary gland Hematoxylin and Eosin (H&E) staining and quantification of (B) alveolar diameter and (C) number. (D) Immunofluorescence staining with Ki-67 proliferation marker (red) combined with E-cadherin (green) and DAPI (blue). (E) Quantification of Ki-67 positive cells. All values are the mean ± SEM (n = 4). Groups with different letters are significantly different (*P*<0.05).

We then evaluated whether*Tph1* ablation affected normal mammary gland histology and cell proliferation. Alveolar size (diameter) was reduced by approximately 50% in *Tph1* ^−/−^ mice, regardless of exogenous 5-HTP administration, when compared to WT alveoli size (*P*<0.001; Figure 1a, b). Alveolar number was not different in *Tph1* ^−/−^ mice compared to WT, but upon 5-HTP administration to *Tph1* ^−/−^ mice, an increase in alveolar number was observed compared to WT (*P*<0.001; Figure 1a, c). The number of proliferating cells decreased about 6-fold in *Tph1* ^−/−^ mice compared to WT (*P* = 0.001), and the number was restored through the administration of exogenous 5-HTP to *Tph1* ^−/−^ mice (Figure 1d, e).

### *Tph1* K.O Mice had Decreased Circulating serotonin and Calcium concentrations during Lactation

**Figure 2:**
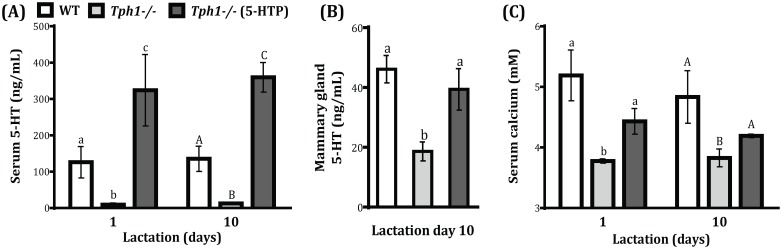
Ablation of Tph1 gene in dams decreased circulating serotonin and calcium on day 1 and 10 of lactation. Wild-type (WT) mouse dams were given saline and Tryptophan hydroxylase knock-out dams given either saline (*Tph1^−/−^*) or daily injections of exogenous 5-HTP (100 mg/kg; *Tph1^−/−^* (5-HTP)), from pregnancy d20 to lactation d10. (A) Serum serotonin (d1 and d10 of lactation) and (B) mammary serotonin concentrations (d10 of lactation). (C) Serum calcium concentration (d1 and d10 of lactation). All values are the mean ± SEM (n = 7). Groups with different letters are significantly different (*P*<0.05).

As expected, *Tph1* ^−/−^ dams were deficient in circulating serotonin compared to WT animals on both d1 and d10 of lactation (*P*<0.038). Administration of exogenous 5-HTP to the *Tph1* ^−/−^ mice restored the circulating serotonin deficiency (*P*<0.012, Figure 2a). Herein, we assessed serotonin concentrations in the mammary gland. *Tph1* ^−/−^ dams had decreased mammary serotonin synthesis (*P* = 0.018) and this deficiency was restored to the WT levels by administering exogenous 5-HTP to the *Tph1* ^−/−^ dams (Figure 2b). In line with previous findings [19] we demonstrated that peripheral 5HT is also necessary to maintain calcium homeostasis during lactation in this experimental model. *Tph1* ^−/−^ mice had decreased serum total calcium concentrations compared to WT mice on d 1 and d 10 of lactation, and this decrease was attenuated by giving *Tph1* ^−/−^ mice exogenous 5-HTP on both days (*P*<0.05; Figure 2c).

### Influx and Efflux of Calcium in the Lactating Mammary Gland is Impaired by serotonin Deficiency

**Figure 3:**
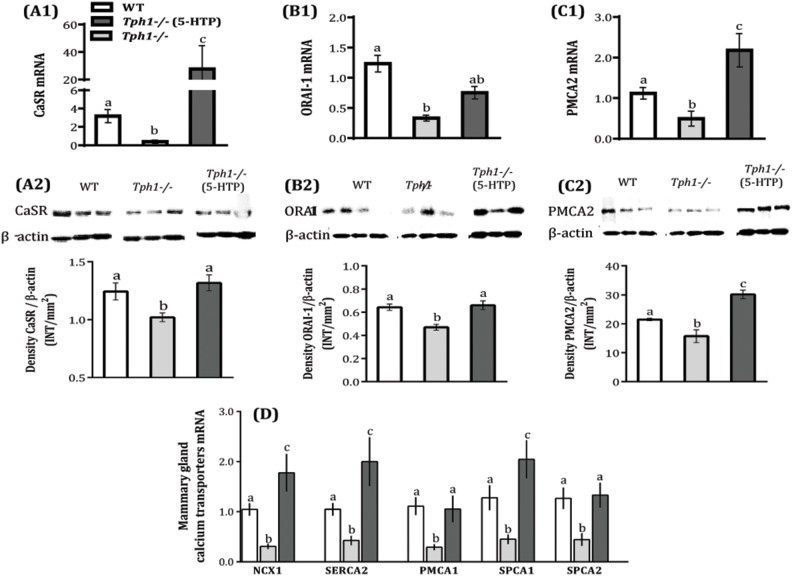
Serotonin deficiency decreases calcium transporters expression in the mammary gland on d 10 of lactation. Wild-type (WT) mouse dams were given saline and Tryptophan hydroxylase knock-out dams given either saline (*Tph1^−/−^*) or daily injections of exogenous 5-HTP (100 mg/kg; *Tph1^−/−^* (5-HTP)), from pregnancy d20 to lactation d10. Mammary gland mRNA and protein expression of *calcium sensing receptor* (CaSR, A1–2), *calcium release-activated calcium channel protein 1* (ORAI-1, B1–2) and *plasma membrane Ca^2+^ ATPase* and *2* (PMCA2, C1–2) on d10 of lactation. (D) Mammary mRNA expression of sodium/calcium exchanger 1 (NCX1), sarcoplasmic endoplasmic Ca^2+^ ATPase 2 (SERCA2), plasma membrane Ca^2+^ ATPase 1 (PMCA1), secretory pathway Ca^2+^ ATPase 1 and 2 (SPCA1 and 2) on d10 of lactation. All values are the mean ± SEM (n = 7). Groups with different letters are significantly different (*P*<0.05).

Lactation is characterized by an extensive trans-cellular flux of calcium, from the basolateral side of mammary alveolar epithelium (blood calcium) into lumen apical side of the cell (milk calcium). We investigated serotonin’s role in the regulation and localization of calcium channels and pumps during lactation. The CaSR has been described as master regulator of systemic calcium metabolism, and is largely responsible for calcium influx into the mammary gland [10]. Mammary gland CaSR mRNA expression was decreased in*Tph1* ^−/−^ mice compared to WT (*P* = 0.007) and was markedly increased after exogenously administration of 5-HTP compared to *Tph1* ^−/−^ and WT (P<0.050; Figure 3 a1). Similarly, mammary CaSR protein level was decreased in *Tph1* ^−/−^ dams compared to WT (*P* = 0.048), and recovered after administering 5-HTP (Figure 3 a2). The store-operated channel ORAI-1, central to calcium signaling in mammalian cells, is restricted to basolateral domains of the mammary plasma membrane and is involved in entrance of calcium to the cell [8]. Herein, ORAI-1 mRNA and protein expression were decreased in the *Tph1* ^−/−^mammary glands compared to the WT (*P*<0.011), and were restored to WT levels after administration of 5-HTP in both cases (Figure 3 b1, b2). PMCA2 is responsible for pumping about 60% of calcium directly across the mammary secretory cells apical membrane [11]. In this study, PMCA2 mRNA and protein expression were decreased in *Tph1* ^−/−^ mammary glands compared to the WT (*P*<0.051). After admiration of 5-HTP mRNA and protein levels in the mammary tissue were increased compared to WT and *Tph1* ^−/−^ (P<0.048; Figure 3 c1, c2).

### Calcium Sequestration and Compartmentalization is altered by serotonin Deficiency

Calcium is an essential and ubiquitous second messenger. Changes in cytosolic calcium trigger downstream signaling cascades which regulate a wide range of cellular functions. Additionally, intracellular calcium levels must be tightly controlled to avoid cytoplasmic calcium toxicity [27]. Intracellular calcium stores are regulated by the actions of active calcium transporters including SPCA1, SPCA2, SERCA2 and PMCA1 [7]. We observed that serotonin deficiency decreases mammary gland mRNA expression of several calcium transporters (*P*<0.006; Figure 3d). Additionally, SERCA2 and SPCA1 mRNA expression increased 2-fold after exogenous 5-HTP was administered to *Tph1* ^−/−^ mice (*P*<0.019; Figure 3d).

### Calcium Sensing and Trafficking is Controlled by Serotonin

**Figure 4:**
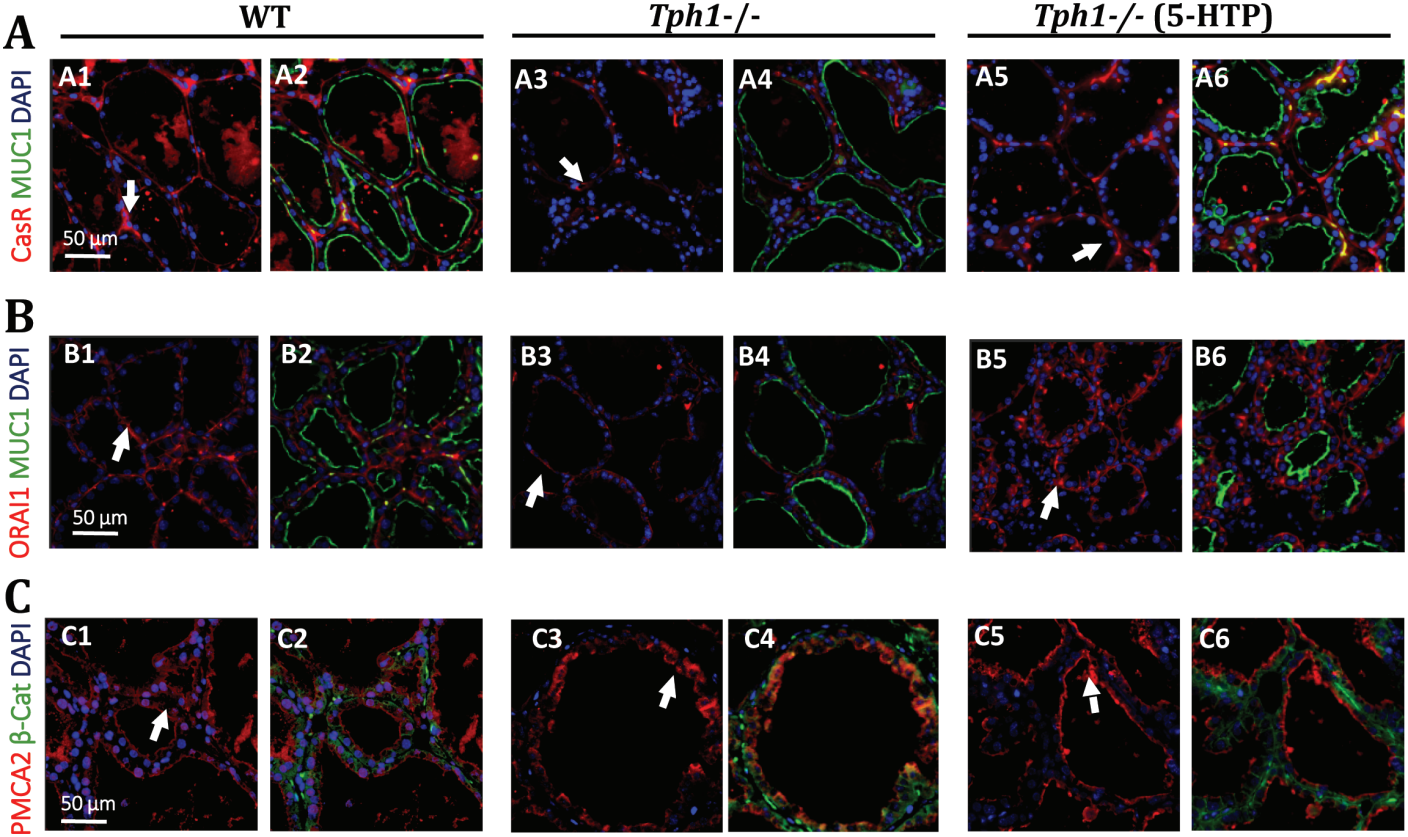
Serotonin deficiency alters the protein abundance and localization of basolateral (CaSR and ORAI1) and apical (PMCA2) calcium transporters in the mammary epithelial cells on d10 of lactation. Wild-type (WT) mouse dams were given saline and Tryptophan hydroxylase knock-out dams given either saline (*Tph1^−/−^*) or daily injections of exogenous 5-HTP (100 mg/kg; *Tph1^−/−^* (5-HTP)), from day 15 of gestation to day 10 of lactation. Representative mammary gland slides (n = 3) immuno stained for calcium sensing receptor (CaSR, red) co-satined with mucin 1 (MUC1, green, apical membrane marker, A1–6), calcium release-activated calcium channel protein 1 (ORAI-1, red) co-satined with MUC1 (B1–6), and plasma membrane Calcium ATPase and 2 (PMCA2, red) co-stained with β-Catenin (β-Cat, C1–6) on d10 of lactation. Blue staining = DAPI (nuclear stain) Green staining = MUC1 or β-Cat. White arrows indicate the localization of the receptors in the alveoli.

The lack of peripheral serotonin in lactating mammary tissue from *Tph1* ^−/−^ mice markedly decreased the abundance of CaSR (Figure 4 a3) compared to the lactating mammary tissue from WT and the *Tph1* ^−/−^ to which 5-HTP was exogenously administered (Figure 4 a1, a5). The WT CasR phenotype is rescued by administering 5-HTP to the *Tph1* ^−/−^ mice (Figure 4 a5–6).

*Tph1* ^−/−^ lactating mammary tissue exhibited less ORAI-1 (Figure 4 b3) compared to WT and *Tph1* ^−/−^ (5-HTP) lactating mammary tissue (Figure 4 b1, b5). PMCA2 is normally present in the apical membrane of the MEC. However, in *Tph1* ^−/−^ deficient dams, we observed a shift of this calcium pump to the intracellular compartments, as seen by the unexpected slight co-localization with the β-Cat basolateral marker (Figure 4 c4). PMCA2 did not colocalize with β-Cat in WT and *Tph1* ^−/−^ (5-HTP) mammary glands (Figure 4 c2, c6).

### Downstream Signaling Pathways of Htr2b in the Mammary Gland

**Figure 5:**
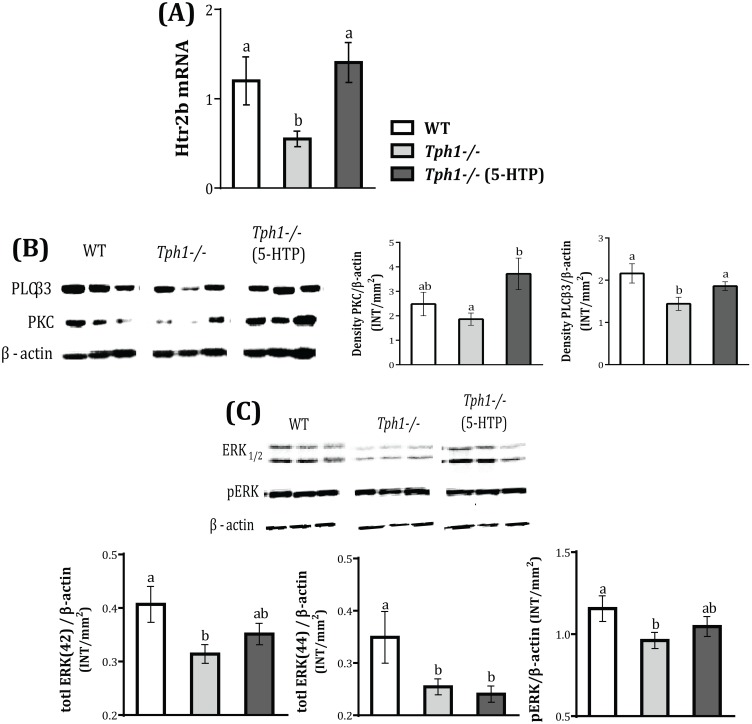
Downstream signaling pathways of the Htr2b receptor in the mammary gland are altered by serotonin deficiency. Wild-type (WT) mouse dams were given saline and Tryptophan hydroxylase knock-out dams given either saline (*Tph1^−/−^*) or daily injections of exogenous 5-HTP (100 mg/kg; *Tph1^−/−^* (5-HTP)), from pregnancy d20 to lactation d10. (A) Mammary gland *Htr2b* mRNA expression (n = 7) and its downstream signaling pathways PLCβ, PKC (B), ERK1/2 and pERK protein expression (C, n = 3) on d10. All values are the mean ± SEM. Groups with different letters are significantly different (*P*<0.05).

We next evaluated the expression of *Htr2b* since the regulation of PTHrP and calcium by serotonin is driven by this receptor sub-type [21]. Mammary gland *Htr2b* mRNA expression decreased in *Tph1* ^−/−^ dams compared to WT (*P* = 0.019), and was rescued by administering 5-HTP to the *Tph1* ^−/−^ mice (*P* = 0.005, Figure 5a). Binding of serotonin to its Gq/11-coupled receptor 2b, activates phospholipase C (PLC), initiating a rapid release of inositol triphosphate resulting in increased intracellular Ca levels, as well as protein kinase C (PKC) and Ras-Raf-Mek-ERK signaling pathways [28], [29]. To determine whether serotonin was acting through Htr2b to elicit downstream signals, we tested and determined that mammary gland PLCβ3 protein levels were decreased in *Tph1* ^−/−^ dams compared to WT (*P* = 0.036) and increased in the 5-HTP dams (*P* = 0.045) reaching similar protein levels to the WT mice (Figure 5b). Mammary gland PKC protein levels did not change with 5-HT deficiency but tended to be increased in the 5-HTP dams (*P* = 0.09; Figure 5b). The mitogen-activated protein (MAP) kinases p44ERK_1_ and p42ERK_2_ are crucial components of the regulatory machinery underlying normal and malignant cell proliferation [30]. Peripheral serotonin deficiency in the *Tph1* ^−/−^ mice decreased ERK_1_ (42 KDa) and ERK_2_ (44 KDa) expression in their mammary glands (*P*<0.048, Figure 5c). Administration of exogenous serotonin was able to partially restore ERK_1_ but not ERK_2_ protein levels in the mammary glands of *Tph1* ^−/−^ mice (Figure5c).

## Discussion

Despite the numerous studies focusing exclusively on the role of neuronal serotonin on mood and behavior, increasing evidence supports the fact that the monoamine serotonin regulates important non-neuronal functions in various organs throughout the body [31], [32]. serotonin is produced by MEC, acting via autocrine-paracrine mechanisms to regulate mammary gland development, milk protein gene expression, milk secretion, and the regulation of tight junctions [13], [16], [17], [26]. Indeed, serotonin regulates maternal calcium levels during lactation through the mammary induction of PTHrP and directly acts on the bone to induce remodeling and support calcium homeostasis [19], [21], [33].

Herein, we demonstrate that non-neuronal serotonin actively participates in MEC calcium transport and in needed for the expression of key calcium transporters and pumps, maintaining alveolar structure and supporting MEC proliferation during lactation. How calcium is transported from the maternal circulation into the MEC and into the lumen of the alveoli during lactation has been extensively investigated [34], [35], and to date the key channels and pumps involved in calcium flux across MECs are known to increase during lactation [7], [36], [37]. However, the mechanisms underlying and potentially regulating these events are only partially understood. Of particular interest was how serotonin affects calcium transport within the mammary gland and the associated molecular mechanisms. We demonstrate that peripheral serotonin deficiency during lactation markedly decreases the expression of key calcium pumps and channels (CaSR, ORAI-1 and PMCA2) both at the protein and transcript level. It is possible that this observation might be an adaptation of the mammary epithelium to the observed decrease in circulating calcium concentrations seen in TPH1 deficient dams.

The lactating mammary gland has the capacity to sense the extracellular calcium concentrations and adjust its secretion into the milk and the secretion of PTHrP into the circulation via the CaSR [36], [38] and therefore the CaSR has been described as a master regulator of systemic calcium metabolism. CaSR-null mice secrete 50% less calcium into the milk [39]. In our study, we demonstrate that lack of serotonin results in decreased mammary CaSR as well as PTHrP (data not shown), but also decreased mRNA expression of other calcium transporters and pumps, including PMCA2 which is responsible for more than 60% of apical transference of Ca to the milk [9]. Even though we did not evaluate calcium content in the milk, we assume a decrease in key calcium transporters will decrease milk calcium. We not only detected decreased PMCA2 (both at the mRNA and protein levels) but also we detected a partial shift in its cellular localization from the basolateral to the intracellular compartment of the mammary epithelium. The anatomic, cellular and subcellular distribution, abundance and localization of a calcium transporter will determine its final function. Potentially, this disruption in the cellular localization of PMCA2 cellular location could be explained by the effects serotonin has on alveolar structure and might be reflected in a decreased efflux of calcium to the milk.

ORAI-1 was recently found to be markedly up-regulated during lactation acting as an important contributor to calcium influx from maternal circulation into the MEC [8], [37]. Our studies indicate that given the absence of peripheral serotonin, ORAI-1 mRNA expression is markedly decreased, in a similar fashion to CaSR, which is also located in the basolateral membrane of MEC. This indicates that the influx of calcium to the mammary gland is compromised due to the deficiency in serotonin levels during lactation. Interestingly, we detected an increased mRNA expression of CasR, ORAI-1, NCX1, SERCA2, PMCA2 and SPCA1 after administration of exogenous 5-HTP to the *Tph1^−/−^* dams, supporting the hypothesis that serotonin may positively affect calcium transport both directly, by increasing its transport and indirectly through CaSR and PTHrP interaction [36], [38]. The results reported in this study support the concept that increasing 5-HT during the periparturient period could be beneficial for improving calcium transport across the MEC in addition to signaling the calcium release from bone during lactation by PTHrP [19], [21]. Abnormal cellular calcium homeostasis, including dysregulation of mammary gland calcium transporters, can cause alterations in normal cell functions such as uncontrolled cellular proliferation and/or cell death which can lead to diseases such as cancer [37], [40].

The major gap in understanding non-neuronal 5-HT’s actions in the mammary gland are in terms of which receptor subtype and downstream molecular pathway is modulating specific physiological responses. The mammary glands of different species express unique serotonin receptor patterns with the Htr7 and 2b receptor subtypes being expressed and conserved within the mammary glands of mice, humans, and cattle [21], [41]. In particular, the receptor subtype Htr2b has been shown to be responsible for serotonin induction of PTHrP in the bovine mammary gland [21]. Herein, the decrease in this receptor subtype in the mammary glands of *Tph1^−/−^* coincides with lower calcium concentrations, therefore confirming its participation in calcium regulation in the murine mammary gland. We also show that serotonin deficiency during lactation provokes a decrease in mammary gland expression of PLC-β and PKC signaling pathways, which are downstream of Htr2b receptor. Peripheral serotonin deficiency decreases mammary expression of ERK_1/2_ as well as its phosphorylation capacity within the MEC. This result is reflected in decreased cell proliferation observed in the *Tph1* K.O mammary glands. The suppression of these signaling cascades might also explain the decreased alveolar number and diameter observed in the *Tph1^−/−^* dams. However, because exogenous administration of 5-HTP to these dams only partially rescued the WT phenotype, we cannot rule out the participation of other extracellular stimuli (i.e. growth factors, cytokines, mitogens, hormones) in these observed outcomes or 5-HT actions through a different receptor subtype. Abnormal regulation of the MAPK pathways has been reported for a wide range of diseases including many cancers, obesity and diabetes [42], therefore these outcomes can have profound implications not only for lactation.

In summary, the results described here demonstrate the necessity of non-neuronal serotonin in achieving or maintaining adequate MEC calcium influx and efflux as well as normal mammary gland morphology and MEC proliferation, all of which are essential for a successful lactation. Our better understanding of the molecular mechanism(s) and pathways involved in the regulation of mammary gland function by serotonin during lactation may open new avenues of research for the potential pharmacological manipulation of this pathway to improve calcium status and therefore reduce the incidence of lactation induced hypocalcemia as well as other disorders that had been positively correlated with serotonin status at the onset of lactation [23]. Additionally, these findings may be relevant for women taking selective serotonin reuptake inhibitors during lactation.
